# Roles of Dietary Amino Acids and Their Metabolites in Pathogenesis of Inflammatory Bowel Disease

**DOI:** 10.1155/2017/6869259

**Published:** 2017-03-14

**Authors:** Xianying Bao, Zemeng Feng, Jiming Yao, Tiejun Li, Yulong Yin

**Affiliations:** ^1^College of Animal Science and Technology, Hunan Agriculture University, Changsha, Hunan 410128, China; ^2^Key Laboratory of Agro-Ecological Processes in Subtropical Region, Institute of Subtropical Agriculture, Chinese Academy of Sciences, Hunan 410125, China; ^3^National Engineering Laboratory for Pollution Control and Waste Utilization in Livestock and Poultry Production, Hunan 410125, China; ^4^Hunan Provincial Engineering Research Center for Healthy Livestock and Poultry Production, Scientific Observing and Experimental Station of Animal Nutrition and Feed Science in South-Central, Ministry of Agriculture, Changsha, Hunan 410125, China; ^5^Hunan Co-Innovation Center of Animal Production Safety, CICAPS, Changsha 410128, China; ^6^Guangdong Wangda Group Academician Workstation for Clean Feed Technology Research and Development in Swine, Guangdong Wangda Group Co., Ltd., Guangzhou, Guangdong 510663, China

## Abstract

Inflammatory Bowel Disease (IBD) is a kind of chronic inflammation, which has increasing incidence and prevalence in recent years. IBD mainly divides into Crohn's disease (CD) and ulcerative colitis (UC). It is hard to cure IBD completely, and novel therapies are urgently needed. Amino acids (AAs) and their metabolites are regarded as important nutrients for humans and animals and also play an important role in IBD amelioration. In the present study, the potential protective effects of AAs and their metabolites on IBD had been summarized with the objective to provide insights into IBD moderating using dietary AAs and their metabolites as a potential adjuvant therapy.

## 1. Inflammatory Bowel Disease Prevalence and Increasing Incidence

Inflammatory bowel disease (IBD) is a kind of chronically multifactorial inflammatory disorder affecting the gastrointestinal tract [1]. It is triggered as a consequence of excessive proinflammatory cytokines production, persistent macrophage activation, and cell death induced by subsequent bacterial or/and viral infections. IBD is the major intestinal health concerns causing severe diarrhea, abdominal pain, weight loss, metabolic disorder, and malabsorption, and it mainly comprises two major forms: Crohn's disease (CD) and ulcerative colitis (UC) [2, 3]. CD and UC have different inflammatory location in the gastrointestinal tract. CD affects all layers of the gastrointestinal tract and is associated with excess expression of IL-12/IL-23 and IFN-*γ*/IL-17, while UC mainly occurs in colon affecting the mucosa with primarily excess production of IL-13 [3, 4]. Currently, the incidence and prevalence of IBD are increasing with time around the world, especially the rate within elderly patients [5]. The developed countries (Europe and North America) present the highest occurrence [6]. In the West, the prevalence of IBD is 37 to 249 cases per 100,000 people for UC and from 26 to 319 cases per 100,000 people for CD [6, 7]. By contrast, Eastern Europe, Asia, and other developing countries have a lower IBD incidence [7–9]. The occurrence of UC in Asian countries is higher than that of CD [10].

The pathogenesis of IBD is complex and still unclear. Mounting evidences suggest that IBD is the consequence of abnormal immune regulation induced by genetic and/or environmental factors (e.g., diet, infection) [1, 11]. Various inflammation mediators including reactive oxygen species and proinflammatory cytokines (NF-*κ*B, cytochrome c, and tumor necrosis factor- (TNF-)-*α*) act as predisposing factors for chronic inflammatory diseases [12]. The typical clinical inflammation of the gastrointestinal tract leads to IBD with excessive production of proinflammatory mediators and loss of the intestinal mucosal barrier integrity. Since there is no complete cure for IBD, the therapies of IBD primarily focus on inducing or maintaining remission and promoting the expression of anti-inflammatory genes [3].

At the present, the conventional treatments of IBD such as biological therapies targeting specific cytokines or pathways and clinical medication have been improved in recent years. Mesalamine, antibiotics, and budesonide are used in patients with mild disease status, while patients with moderately severe IBD take systemic corticosteroids, immune-modulators (thiopurine analogues, azathioprine (AZA), 6-mercaptopurine (6-MP), and methotrexate), and anti-TNF*α* agents (infliximab, adalimumab, and certolizumab pegol) [13]. However, proper administration selection becomes a common clinical dilemma with those different kinds of medicines. The conventional treatments in severe IBD have a short-term favorable prognosis, while there is still a challenge in the development of alternative therapies character with low-risk and long-term outcomes [14]. Besides, treatments mentioned above are proved to have limited efficacy, adverse effects, drug interactions, and potential toxicity [15, 16]. The use of AZA and 6-MP in IBD patients leads to serious adverse drug reactions such as hepatotoxicity, pancreatitis, and gastrointestinal disturbances [17]. Immunosuppressive and anti-TNF therapy in IBD cause dermatological adverse effects, including skin infections, drug hypersensitivity, psoriasis, eczema, and nonmelanomatous skin cancer [18, 19].

In most cases, patients suffering IBD usually face long duration which becomes a considerable economic burden. With the development of medical and life science, a large number of new strategies are provided to treat IBD. Functional nutrients have aroused growing interest, which help prevent or remit malnutrition, moderate the mucosal immune response, and benefit intestinal homeostasis [20–22]. Essential nutrient has the potential to ameliorate restore redox balance and inflammation in the gastrointestinal tract showing the possibility of nutrients in IBD treatment [3]. Part of the nutrients that benefit the management of IBD had been listed in Table 1.

## 2. Amino Acids: Application in Inflammatory Bowel Disease Therapies

Among IBD benefit nutrients, amino acids (AAs) act as the key regulatory factor in metabolic pathways controlling and have important effects on keeping the intestinal health. AAs are considered as the building blocks for protein synthesis and also play major roles in other functions, such as cell signaling, gene expression, intracellular protein turnover, maintenance, reproduction, oxidative stress, and immunity [46, 47]. Systemic inflammation can cause malnutrition symptoms and general glutamine (Gln) deprivation which is associated with depression, muscle loss, and emotional fatigue [48]. Both UC and CD disturb AAs metabolism in serum and plasma by increasing levels of isoleucine (Ile) (and its first degradation product 3-methyl-2-oxovalerate), methionine (Met), lysine, glycine (Gly), arginine (Arg), and proline (Pro), while decreasing levels of valine, tyrosine, and serine [49]. Some of the increased AAs were also reported to be increased in fecal extracts [50], while Ile and leucine (Leu) have apparently low concentrations in colonic mucosa of active IBD [51]. Met is an essential amino acid and a precursor of homocysteine, a metabolite shown highly elevated in both plasma and colonic mucosa from IBD patients [52].

AAs also have trophic and cytoprotective effects on health in humans and animals [53, 54]. T cells are regarded as central effectors of the adaptive immune system. T helper (Th) cells (Th1, Th2, and Th 17 cells) differentiate from native CD4^+^ T cells and are involved in the pathogenesis of several inflammatory immune-mediated disorders such as producing different cytokines in immune responses [55]. CD is a Th1-type T cell-mediated inflammation while UC is a Th2-type T cell-mediated inflammation [4]. AAs directly serve as a fuel source for T cells and are considered to have influence on shaping T cell-mediated immune responses [56]. Additionally, the signaling central integrator of environmental stimuli for the regulation of T cell activation and differentiation is mammalian target of rapamycin (mTOR) [57, 58]. mTOR have two distinct protein complexes, mTOR complex 1 (mTORC1) and mTOR complex 2 (mTORC2). In T cells, AAs work as signaling molecules with mTORC1 acting as a key mediator. AAs regulate the intracellular localization and activation of mTORC1 by the lysosome-based signaling system composed of Ras-related GTPases (Rags) and Regulator v-ATPase, GAP activity towards Rags, and folliculin complexes [59, 60]. AAs could protect whole body and muscle from protein loss* via* mTOR activation and downstream signaling to protein synthesis through mTORC1 in the acute phase of inflammation [61, 62]. Leu-enriched diet accelerated recovery from muscle damage by alleviating excessive expression of proinflammatory cytokine and preventing invasion of inflammatory cells into muscle [63]. Otherwise, general controlled nonrepressed (GCN2) kinase is a key orchestrator of the integrated stress response which senses AAs depletion. Acute AAs starvation in mice protects the symptoms of colitis, limits Th17 cells, and suppressed IBD via a GCN2-dependent mechanism, providing another mechanism of AAs in regulating IBD [64].

In conclusion, growing evidence shows that the anti-inflammatory activities of tryptophan (Trp), Gln, Met, cysteine (Cys), and Arg have been well established, suggesting a therapeutic role of AAs in IBD, which were listed in Table 2. It is necessary to illustrate the biological activity of specific immunomodulatory AAs in IBD.

### 2.1. Tryptophan

Trp is an essential AA for human and animals and plays an important role in inflammatory regulation beyond building block for proteins synthesis [65]. The serious Trp concentration shows a highly inverse correlation with disease activity in IBD patients [66]. Dietary supplementation of Trp can reduce IBD through its immune-regulatory metabolites [35, 36]. In a porcine model of dextran-sodium sulfate- (DSS-) induced colitis, Trp administration at 80% of the daily recommended intake could ameliorate colitis clinical symptoms, improve histological parameters and intestinal permeability, reduce the expression of proinflammatory cytokines, and increase the expression of proapoptotic genes, which is important for maintaining gut homeostasis [35]. Trp presents strong anti-inflammation activity by triggering calcium-sensing receptor (CaSR) activation in intestine [67], which is a sensing receptor for dietary nutrients in the gastrointestinal tract to maintain mucosal immune homeostasis. Trp treatment allosterically activate CaSR which significantly reduces TNF-*α*-induced interleukin- (IL-) 8 secretion indicating a novel therapy in intestinal inflammation [67].

In addition, Trp exerts anti-inflammatory function via the metabolites including serotonin (5-hydroxytryptamine, 5-HT) and melatonin (MT). 5-HT is an important compound derived from Trp which serves as a neurotransmitter and inhibiter of the production of inflammatory cytokines and superoxide [46]. MT is abundant in gastrointestinal tract [68, 69] and has a positive impact on IBD with no or negligible side effects due to regulation of macrophage activity, reduction of cell migration and myeloperoxidase activity, and inhibition of NF-*κ*B and TNF-*α* expression [36, 68, 70]. MT added to omeprazole treatment obviously accelerates chronic gastroduodenal ulcers over the obtained with omeprazole alone [71]. Additionally, in mice with DSS-induced colitis, MT exerts anti-inflammatory effects by alleviating the severity of mucosal injury and recovering the expression of IL-6, IL-17, and adiponectin [72].

### 2.2. Glutamine and Glutamate

As an abundant AA in the blood and tissues, Gln is mostly used as nitrogen source and/or alternative energy fuel [73]. In various clinical situations, appropriate exogenous Gln supply is safe and can beneficially contribute to diminishing risks of high-dose chemotherapy and radiation [74]. It has been additionally implicated as an immunomodulatory nutrient [75] and has pharmacological function in the IBD treatment [76]. Gln plays a key role in maintaining the integrity of the intestinal mucosa and has been shown to reduce inflammation and relieve CD [77]. In an UC mouse model, dietary Gln supplementation combined with dietary fiber and oligosaccharide has suppressive effects on mucosal damage [78]. A Gln diet replacing 25% of the total nitrogen decreases the expression of chemokine and endothelial adhesion molecules via suppression of T cell migration in mice [37]. These experimental data suggest Gln as a potential nutrient in protecting intestinal integrity and modulating immunity.

Glutamate (Glu) is produced from Gln with the catalysis of glutaminase and generally plays roles in protein synthesis and energy metabolism. Dietary Glu can also function as a signal to regulate the gastrointestinal tract via the gut-brain axis [9]. As a precursor of glutathione (GSH), Arg and Pro, Glu is of critical importance in intestinal metabolism and physiology [53]. Microinjection of Glu into the hypothalamic paraventricular nucleus in UC rats significantly increases the cell proliferation and antioxidant levels and decreases apoptosis and the expression of proinflammatory factors in the colonic mucosa [38]. Poly-*γ*-glutamate (P-Glu) significantly reduced histopathological evidence of injury, attenuated DSS-induced blood vessel densities, and attenuated DSS-induced expression of VEGF-A and its receptor in C57BL/6 mouse colitis model. P-Glu has potential application in conditions marked by inflammatory-driven angiogenesis and mucosal inflammation [79]. These findings above indicate that dietary supplementation of Gln and Glu is of functional and nutritional importance in intestinal mucosal growth and gut inflammation.

### 2.3. Sulfur Amino Acids

Sulfur amino acids (SAAs) mainly contain Met, Cys, and cystine. SAAs metabolism mainly takes place in the gastrointestinal tract. Dietary deficiency of SAAs will suppress intestinal mucosal growth, reduce intestinal epithelial cell proliferation, and increase intestinal oxidant stress in piglets [80].

As essential AA, Met has been considered as the first and second or third limiting AA in poultry and nursery pigs, respectively. Piglets fed the diet supplemented with Met present increased growth performance and exhibited improved integrity and barrier function of the small-intestinal mucosa [39]. Dietary supplementation with Met metabolites also can affect the susceptibility to colitis, reduce inflammation and tissue injury, and decrease the expression of multiple inflammatory genes in mice [81]. It is also interesting that Met (twice NRC recommendation) combined with fish oil (2.5%) can enhance immune response in IBD challenged broiler chickens which may be a novel treatment in IBD therapy for poultry [82].

Cys is a nonessential AA playing roles in protein metabolism and is regarded as the key factor in the synthesis of GSH. Dietary supplementation with* N*-acetyl-L-cysteine improves the clinical symptoms and decreases the chemokines without any side effects [83]. Cys administration can attenuate DSS-induced weight loss and intestinal permeability, decrease the expression of proinflammatory cytokines, and restore susceptibility of activated immune cells to apoptosis, indicating Cys supplementation as a novel therapy for IBD [40]. In addition, *γ*-glutamyl cysteine treatment can ameliorate DSS-induced clinical signs and histological damage* via* activating CaSR [84].

Besides, GSH, taurine (Tau), and hydrogen sulfide (H_2_S), the products of catabolism of SAAs, play major roles in anti-inflammation and antioxidant system [46]. Tau is regarded as an antioxidant and membrane stabilizer against oxidative stress and inflammation by inhibiting chemokine secretion from intestinal cells [85, 86]. Moreover, the cellular metabolite taurine chloramine of Tau in the rectal shows anti-inflammatory property on IBD via inhibition on NF-*κ*B activity [44, 87]. Tau treatment exerts beneficial effects in rats with 2,4,6-trinitrobenzene sulphonic acid- (TNBS-) induced colitis with decreasing inflammatory reactions and apoptosis [88]. H_2_S is a signaling molecule and a gaseous mediator that exhibits several anti-inflammatory activities and contributes to mucosal protection [89–91]. One of the possible mechanisms of H_2_S in the resolution of IBD is proved to be mediated via stabilization of hypoxia-inducible factor-1*α* [92].

### 2.4. Arginine

Arg is a semiessential AA that has protective effects against oxidative stress. As the substrate for nitric oxide (NO) synthesis, amino acid Arg is reported to be therapeutic in wound healing and has potent anti-inflammatory properties as a mediator of autoimmune diseases [93–96]. Otherwise, exogenous Arg is associated with antiapoptotic effects on the rat intestine and useful in the treatment of intestinal ischemia/reperfusion injury [97]. Dietary supplementation with Arg can improve the immune status of humans and animals and has the potential to be used to supplement current treatments for IBD [12]. Serum Arg concentration is a useful biomarker of UC disease severity [98]. Further, in a DSS-induced fulminant colitis murine model, treatment with hepatocyte growth factor and Arg can decline associated symptoms such as pain and diarrhea [99]. Arg is absorbed and transferred by cationic AA transporters (CAT) in intestine. DSS-induced inflammation reduced the expression of CAT2 in colonic and Arg uptake with body weight loss, reducing colonic permeability. Supplementation with Arg markedly attenuates the clinical parameters above and reduces the expression of proinflammatory cytokine and chemokine [42]. Arg might reduce the inflammation associated with AA-induced colitis through the NF-*κ*B/nitric oxide pathway [100]. NO participates in nutrient metabolism and exerts protective effects against IBD including inhibition of macrophage activation and proinflammatory cytokine levels [101].

### 2.5. Other AAs

Besides the functional AAs above, other AAs have been reported to possess anti-inflammation functions to some extent. In addition to acting as an important precursor for the biosynthesis of GSH, Gly is proved to ameliorate diarrhea and body weight loss in TNBS induced colitis in the rats, indicating that Gly may be a useful immunomodulating nutrient for the treatment of IBD [43]. Histidine has proven to be a novel therapeutic agent for CD by inhibition of NF-*κ*B activation, downregulating proinflammatory cytokine production in IL10^−/−^ mice [41]. Ergothioneine (EGT) is a natural water-soluble amino acid which can be derived from mushroom or synthesized by nonyeast fungi [102, 103]. In ultraviolet-B-irradiated mice, the administration of EGT inhibited the UV-B-induced inflammatory responses and DNA halogenation, showing the modulatory effects of EGT in inflammation [103].

## 3. Future Perspectives and Challenge

Lacking of effective medical therapies for IBD makes it of utmost importance to find alternative therapeutic strategies [3]. AAs can relieve intestinal inflammation through regulation of proinflammatory cytokines suggesting a possible approach to IBD treatment. However, further investigations and clinical studies are needed to fully understand the therapeutic mechanism and potential of AAs in preventing inflammation in both humans and animals.

## Figures and Tables

**Table 1:** Studies on dietary nutrients supplementation for the management of IBD.

Nutrients	Primary components	Chemical structure	Functions	Ref.
Corabion	A mixture of vitamin C, vitamin E, *ω*3-PUFAs (EPA and DHA), and Arg		Reduction of DAI, neutrophil recruitment, oxidative stress, proinflammatory cytokines, and E-cadherin internalization; attenuation of colon shortening and tissue damage	[23]

Pomegranate extract	Ellagic acid	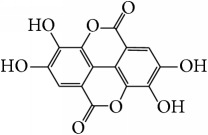	Reduction of MPO activity, TNF-*α* levels, (COX-2), iNOS overexpression, and MAPKs phosphorylation; preventing NF-*κ*B translocation	[24, 25]

Krill oil	*ω*3-PUFAs and phospholipids		Reduction of DAI, HCS, colon length, and protein oxidation markers; improvement of Pparg1*α* and (PG)E3 expression	[26]

Tetradecylthioacetic acid	An artificial 16-carbon fatty acid with a sulphur-substitution in the *β*-position	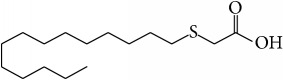	Reduction of colonic oxidative damage and colon wall thickness; improvement of expression of Pparg1*α*; inhibition of the production of inflammatory cytokines (TNF-*α*, IL-1*β*, and IL-6)	[27]

Fibre	Nonstarch polysaccharides, resistant oligosaccharides, analogous carbohydrates, and lignin		Maintain remission and reduce lesions of the intestinal mucosa	[28, 29]

Anthocyanins	Natural agents derived from strawberry, blueberry, barberry, and other plants	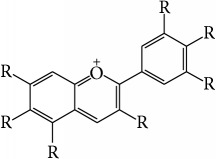	Cytoprotection; remission of oxidative stress and inflammatory cytokines; suppression of cellular signaling pathways of inflammatory processes	[30]

*α*-Linolenic acid	*ω*3-PUFA: plant-derived oil	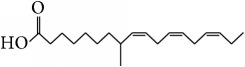	Inhibition of the production of IL-6 and TNF-*α*; reduction of cell apoptosis, intestinal permeability, and bacterial translocation; improving histological repair	[31, 32]

FAAH inhibitors	PF-3845	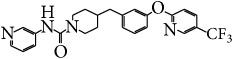	Possess anti-inflammatory effect in TNBS-induced colitis in mice; alter the levels of endocannabinoids	[33]
	FAAH-II	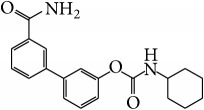	Inhibition of inflammatory miRNAs and cytokines; reduction of the number of activated T cells, the frequency of macrophages, and neutrophils in the colon	[34]

EPA, eicosapentaenoic acid; DHA, docosahexaenoic acid; (COX-2), cyclooxygenase-2; iNOS, inducible nitric oxide synthase; MAPKs, mitogen-activated protein kinases; MPO, myeloperoxidase; NF-*κ*B, nuclear transcription factor kappa B; PUFAs, polyunsaturated fatty acids; DAI, disease activity index; HCS, histological combined score; Pparg1a, PPAR-g coactivator 1*α*; (PG)E3, prostaglandin-E3; IL-1*β*, interleukin-1*β*; PUFA, polyunsaturated fatty acids; FAAH, fatty acid amide hydrolase.

**Table 2:** Application of partial AAs in inflammatory bowel disease therapies.

AAs	Dose	Administration method	Models	Major functions and effects	Ref.
Tryptophan	88 mg/kg·BW·day	Intragastric infusion	Piglets	Amelioration of clinical symptoms; reduction of gut permeability and cell apoptosis; inhibition of the production of inflammatory cytokines (TNF-*α*, IL-6, IFN-*γ*, IL-12p40, IL-1*β*, and ICAM-1)	[35]

Melatonin	20 mg/kg·BW·day	Intragastric infusion	Rats	Antioxidant; inhibition of the production of inflammatory cytokines (TNF-*α*, COX-2, SOD, and NF-*κ*B); accelerating healing of gastric ulcer	[36]

Glutamine	25% of the total nitrogen	Dietary	Mice	Anti-inflammation; reduction of expression of PSGL-1, LFA-1, and CCR9 by Th cells	[37]

Glutamate	12 *μ*g Glu/0.3 *μ*L saline	Microinjection	Rats	Neurotransmitter; inhibition of T-cell response and inflammation	[38]

Methionine	0.12% L-Met	Dietary	Piglets	Protection of the small-intestinal mucosa	[39]

Cysteine	144 mg/kg·BW·day	Intragastric infusion	Piglets	Reduction of intestinal permeability and cell apoptosis; inhibition of the local expression of inflammatory mediators (IL-6, TNF-*α*, IL-12p40, and IL-1*β*)	[40]

Histidine	Not mentioned	Dietary	Mice	Reduction of histologic damage, colon weight, IL-6, and TNF-*α* production; inhibition of NF-*κ*B	[41]

Arginine	1% (wt/vol) solution	Drinking water	Mice	Improvement of the clinical parameters and body weight loss; reduction of the colonic permeability; reduction of the proinflammatory cytokine and chemokine expression	[42]

Glycine	5% Gly	Dietary	Rats	Diarrhea amelioration; prevention of the increases of IL-1*β* and TNF-*α*	[43]

Taurine	30 mM	Rectal administration	Rats	Anti-inflammation; inhibiting NF-*κ*B activity	[44]
	2%	Drinking water	Mice	Inhibitory effects on the secretion of MIP-2; cytoprotective functions on the epithelial barrier	[45]

TNF-*α*, tumor necrosis factor-*α*; IL, interleukin; IFN-*γ*, interferon-*γ*; ICAM-1, intracellular adhesion molecule-1; PSGL-1, P-selectin glycoprotein ligand-1; LFA-1, leukocyte function-associated antigen-1; CCR9, C-C chemokine receptor type 9; MIP-2, macrophage inflammatory protein 2.
